# High abundances of crustose coralline algae inside cryptic coral habitats linked to coral reef functioning

**DOI:** 10.1098/rsos.241971

**Published:** 2025-08-20

**Authors:** Anna Rothstein, Orion S. McCarthy, Sophie Luu, Emily L. A. Kelly, Philip Heller, Stuart Sandin, Jennifer E. Smith, Maya S. deVries

**Affiliations:** ^1^ Department of Biological Sciences, San José State University, San Jose, CA, USA; ^2^ Scripps Institution of Oceanography, University of California San Diego, La Jolla, CA, USA; ^3^ World Economic Forum, Cologny, Geneva, Switzerland; ^4^ Department of Computer Science, San José State University, San Jose, CA, USA

**Keywords:** turf algae, benthic community composition, coral crevice, large-area imagery

## Abstract

Cryptic habitats on coral reefs consist of crevices, tunnels, and holes that are estimated to comprise 30%–75% of the total reef surface area. These large habitats are vastly understudied because they are often difficult to access. Crustose coralline algae (CCA) are thought to perform important ecological functions, including calcium accretion, both inside cryptic habitats and on the exposed (top) reef. Using GoPro cameras, we surveyed 250 coral crevices from 13 reefs on West Maui, Hawai‘i. We compared relative CCA cover between crevice microhabitats and the well-studied top-reef and identified abiotic and biotic factors that correlated with CCA abundance. We found that crevices had approximately 3.1 times more CCA cover than the top-reef and that CCA cover was highest on crevice ceilings and entrances, which had low sediment and macroalgal cover, compared to the back walls of crevices, which had higher sediment abundance. These results suggest that crevice openings and ceilings are key microhabitats for CCA and therefore may also be important for reef building and stabilization. Thus, these findings help establish functional links between coral reef structural complexity, cryptic habitats, and reef resilience, and highlight the importance of studying cryptic habitats to inform approaches to coral reef conservation.

## 1. Introduction

Coral reefs occupy less than 1% of the Earth’s surface area, yet these ecosystems are among the most biodiverse in the world, serving as crucial refugia and nursery grounds for 25%–33% of all marine life (1–9 million species) [1–3]. Coral reefs form complex, three-dimensional structures with many tunnels and crevices that provide microhabitats for a diverse community of fishes and invertebrates; yet most biodiversity estimates are based on the exposed outer surfaces of the reef [4]. As a result, the biological, ecological, physical and chemical processes that occur within the cryptic spaces of coral reefs remain understudied, because they are difficult to access nondestructively [4–7].

As biogenic structures, coral reefs continuously experience both calcium accretion, which builds the reef framework, and biological and mechanical erosion. Accretion and erosion processes shape structural complexity by creating small tunnels and crevices throughout the reef framework [8]. These cryptic spaces comprise a substantial portion of the three-dimensional coral reef framework, as their surface area may exceed that of the exposed reef by 2−3 times [9].

Cryptic reef habitats provide a foundation for uniquely diverse communities and are probably important contributors to coral reef resiliency worldwide [3,10]. A relatively small body of literature has revealed that reef crevices are dominated by turf, fleshy, and encrusting algae, sessile invertebrates (e.g. encrusting sponges, corals, gastropods and other molluscs, annelids), motile invertebrates (e.g. gastropods, crustaceans, echinoderms) and cryptobenthic fishes [5,7,11]. Emerging research has also emphasized that, while there may be over one million species residing inside the coral reef framework, only about 100 000 of those species have been described, with many cryptic species not represented in current biodiversity estimates [3,7,10,12].

Many of these cryptic species probably aid in maintaining reef structural integrity and nutrient cycling, allowing reefs to grow in naturally low-nutrient systems [11,13–16]. For example, one key contributor to ecosystem functioning is crustose coralline algae (CCA), a group of encrusting, calcareous red algae in the family Corallinaceae. For decades, scientists have associated the presence of CCA with healthy coral reef ecosystems [17,18]. While living, CCA can induce larval settlement of reef-building stony corals through external biochemical signalling [19]. CCA species have also been shown to help alleviate competition for space between corals and fleshy macroalgae on the exposed top-reef [18] through the shedding of their upper surface layers that have macroalgal growth on them and the secretion of antifouling compounds that impede attachment and growth of macroalgae [20–22].

Given that corals comprise only a portion of the reef framework [23], CCA has been shown to contribute to accretion and stabilization of the reef matrix as well [17,24,25]. CCA binds carbonate sediments, cements reef components and fills interstitial spaces, thereby improving reef stability and structural integrity [17]. Both living and dead CCA contribute to these processes, but live CCA is critical for consistent accretion of carbonate material [26]. In fact, estimates of carbonate production from CCA suggest that CCA may be contributing more to carbonate production on reefs than corals, particularly following disturbances that yield mass mortality events of corals [24].

This understanding of CCA function is primarily based on data from the exposed (top) of the reef and from efforts to simulate cryptic spaces to determine calcium carbonate accretion rates inside reefs ([27] and references therein). Calcification accretion units (CAUs), for example, that use stacked settlement tiles to simulate cryptic spaces help standardize measurements of net accretion of carbonate on reefs. Price *et al*. [28] found 27%–62% cover of CCA on CAUs in the Northern Line Islands (Palmyra, Kingman and Jarvis) and then estimated reef construction and degradation rates from those estimates of CCA per cent cover inside reefs [27–29].

While CCA is assumed to contribute to stabilization within the cryptic spaces of a reef, this assumption has never been rigorously tested *in situ *to our knowledge. To understand the extent to which CCA inside reefs contributes to reef function (e.g. stabilization, coral recruitment, recovery post-disturbance), it is critical to first determine the distribution and abundance of CCA in these cryptic habitats. Various CCA species are adapted to low light conditions, allowing them to compete effectively for space and colonize crevice environments in high abundances [17,26,30–32]. Thus, as with some low-light adapted corals [33], the distribution patterns of CCA may be governed by light availability and depth within a crevice. The outcome of CCA competitive dominance in crevices may ultimately result in stabilization of reef structure from within the crevices.

Sediment dynamics can also restrict suitable habitat for CCA growth, as excessive sediment cover can suffocate CCA [34]. For example, on the exposed reef, CCA abundance typically peaks on the outer shelf and decreases towards the inner shelf, probably owing to higher sedimentation loads in shore [34]. Crevices and tunnels inside the reef accumulate sediments from various sources, such as bio-eroded material from boring organisms, faecal matter and terrestrial inputs, such as stormwater run-off [35]. Crevices are more likely than the exposed reef to retain these sediments owing to reduced water flow and flushing [5]. Sediment dynamics are also significantly influenced by one of CCA’s main competitors, turf algae, which form mats of small filamentous algae that trap sediments [36]. Turf algae are less successful than CCA in low-light conditions [30,31] but are thought to bind 87% of all sediment on and within reefs [37,38]. Consequently, it is essential to understand the dynamics of sediment-laden turf within reefs, especially in cryptic habitats that may already harbour disproportionately higher sediment loads.

Using low-cost, accessible methodology and equipment, we determined the distribution and abundance of CCA inside reefs. Specifically, we addressed three focal questions: (i) what is the abundance and distribution of CCA in reef crevices; (ii) do abiotic (light, crevice depth and sedimentation) and biotic (turf algae abundance) factors help govern CCA distribution; and (iii) how does CCA abundance inside the reef compare to CCA abundance on the well-studied exposed top-reef (hereafter referred to as the top-reef)? Answers to these questions will broaden our understanding of how cryptic habitats contribute to holistic reef functioning.

## 2. Methods

### 2.1. Site description

**Figure 1.**
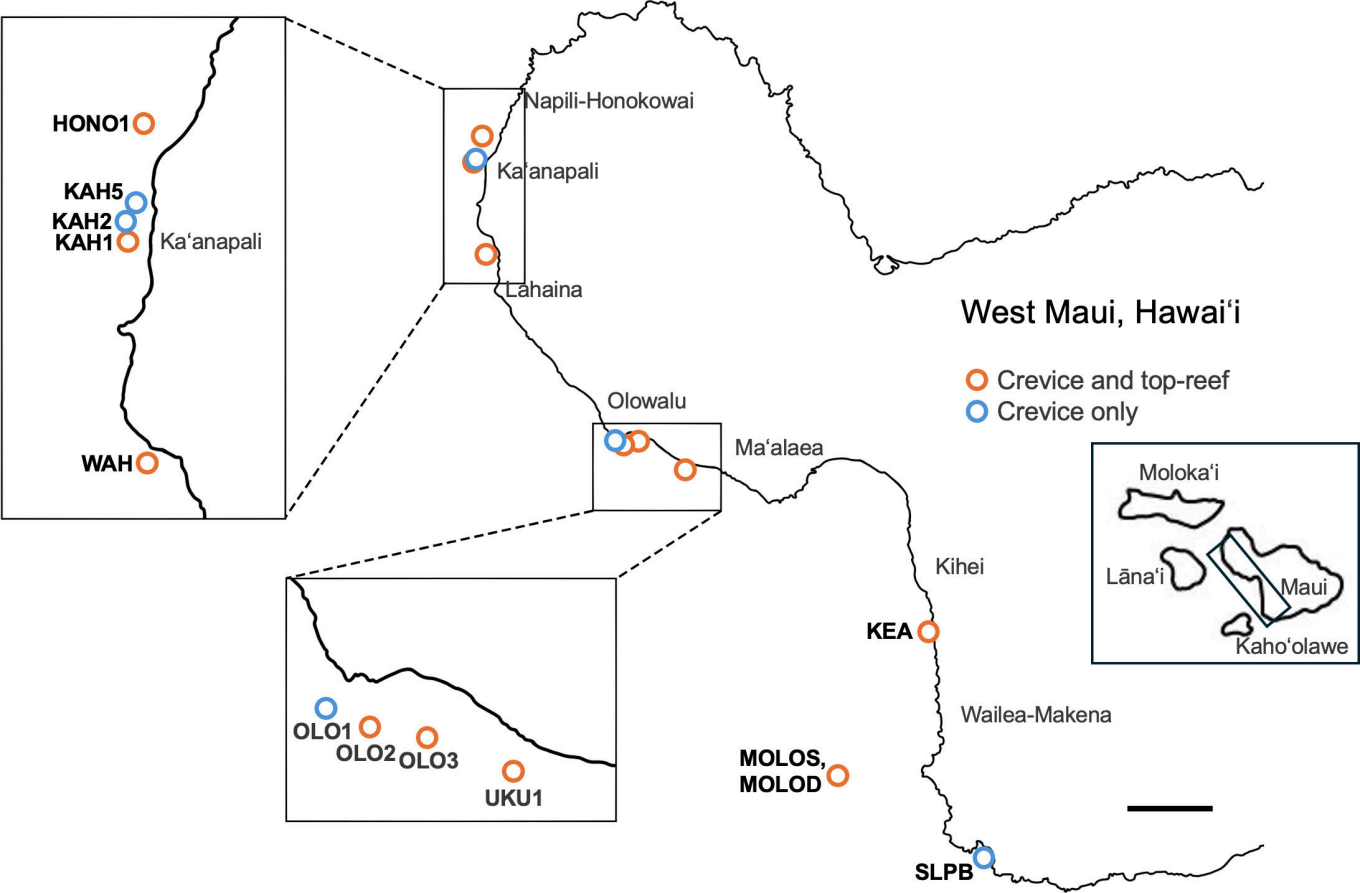
Thirteen sites surveyed along the coast of West Maui, Hawai‘i. Orange circles indicate the sites with both crevice and top-reef data (listed from north to south: Honokowai 1 (HONO1), Kahekili 1 (KAH1), Wahikuli (WAH), Olowalu 2 (OLO2), Olowalu 3 (OLO3), Ukumehame 1 (UKU1), Keawakapu (KEA), Molokini Shallow (MOLOS), Molokini Deep (MOLOD)). Blue circles represent sites with only crevice data (Kahekili 2 (KAH2), Kahekili 5 (KAH5), Olowalu 1 (OLO1), South La Perouse Bay (LAPS)). Site names follow standardized naming conventions of the 100 Island Challenge. Scale bar = 1 km. Inset of Maui and surrounding islands depict the islands once connected as the single island of Maui Nui. West Maui is highlighted in the rectangle. Illustration by Nhi Ly.

From 13 to 23 August 2022, 250 reef crevices were surveyed at 13 sites that ranged in depth from 5 to 12 m (with seven of the sites at 10 m) along the leeward coast of West Maui, Hawai‘i (permit: SAP 2022-62, Hawai‘i Division of Aquatic Resources). Maui is part of a complex of islands that were once connected as the single island of Maui Nui and are now separated by shallow water channels. Thus, leeward West Maui experiences reduced wave exposure and therefore higher coral cover compared to other Hawaiian Islands making this an excellent location for understanding CCA cover inside reefs compared to exposed top-reef sites (reviewed in [39]; figure 1). For example, coral cover on the exposed top-reef of West Maui ranges from 15% to 60%, and fine-scale structural complexity is generally high compared to the rest of the Hawaiian archipelago [40].

Additionally, the top of the reef has been surveyed annually at these 13 sites in established 10 x 10 m plots since 2014 through the 100 Island Challenge long term monitoring programme at Scripps Institution of Oceanography, University of California, San Diego ([39,41,42]; figure 1). While the 100 Island Challenge surveys reefs globally, West Maui is of particular interest, because it has been closely monitored though this programme in collaboration with the Hawai'i Division of Aquatic Resources (reviewed in [39,41,42]). We therefore had access to fine scale data of CCA cover on the top-reef for eight of the 13 sites that were collected in conjunction with the crevice data and that we could use to compare to CCA abundance inside reefs (figure 1).

Benthic top-reef communities primarily consisted of corals, turf algae and CCA. Sites were dominated by a mixture of branching and massive *Porites* spp. and *Pocillopora* spp. as well as branching and encrusting *Montipora* spp. [39]. Sites dominated by branching coral species (*Porites compressa* and *Pocillopora* spp.; sites: HONO1, KEA, OLO2; definitions of site abbreviations, see figure 1) tended to be more structurally complex, with larger, more accessible crevices compared to sites dominated by plating (*Montipora capitata, Montipora patula*; sites: MOLOS, OLO3; figure 1) and massive corals (*Porites lobata*; sites: KAH1, UKU, WAH; figure 1), which were less structurally complex and had smaller crevices that were more difficult to access [40]. Abundances of coral recruits were relatively low at all sites compared to elsewhere in Hawai‘i [43,44], as were herbivorous fishes and sea urchins [45], including those within the Kahekili Herbivore Fisheries Management Area (figure 1; sites KAH1−5), which was established in 2009 as a no-take area for herbivorous fishes [46].

### 2.2. Crevice selection and benthic cover estimates

At each site, crevices were surveyed on SCUBA within the standard 10 × 10 m plots established by the 100 Island Challenge. Mean ± s.d. of 19 ± 3 (range = 14–24, 250 total) crevices were documented per site. If the number of crevices within a 10 × 10 m plot that could accommodate our surveillance equipment was insufficient, researchers would sample crevices outside of the plot but close to the plot perimeter.

Crevices were defined as holes and grooves within the substrate that were accessible from one direction, following [47]. We restricted crevice selection to those accessible from horizontal reef substrate (as opposed to reef walls) in an effort to reduce dramatic differences in light availability between crevices on different parts of the reef. Entrances to most crevices had overhangs and were therefore entered vertically (electronic supplementary material, videos S1 and S2). Crevices were selected if their dimensions, in the judgement of the investigator, were at least 8 cm × 10 cm and at most 60 cm × 100 cm. The minimum size of crevices was notably limited by the camera set-up used to document the benthos (described below), which was 8 cm × 10 cm.

To document benthic cover inside reefs, we used GoPro Hero 8 cameras (GoPro, San Mateo CA, USA, Model CHDHX−801) with a Light in Motion Sidekick Duo S/F attachment (Light and Motion, Marina CA, USA, Model 856-0575-A) on a hand-held, telescopic extender (e.g. selfie stick; camera set-up hereafter referred to as the camera). We used a focal length of 15 mm for filming. Once inside a crevice, we faced the camera forwards into the crevice and recorded while moving the camera until it reached the back wall of the crevice or until the maximum length of the selfie stick was reached (100 cm). Once the back wall was captured, the camera was turned to face the ceiling of the crevice at the same crevice depth as the wall video. We did not document the floor of crevices due to sedimentation obscuring benthic cover in this part of crevices (electronic supplementary material, videos S1 and S2).

**Figure 2.**
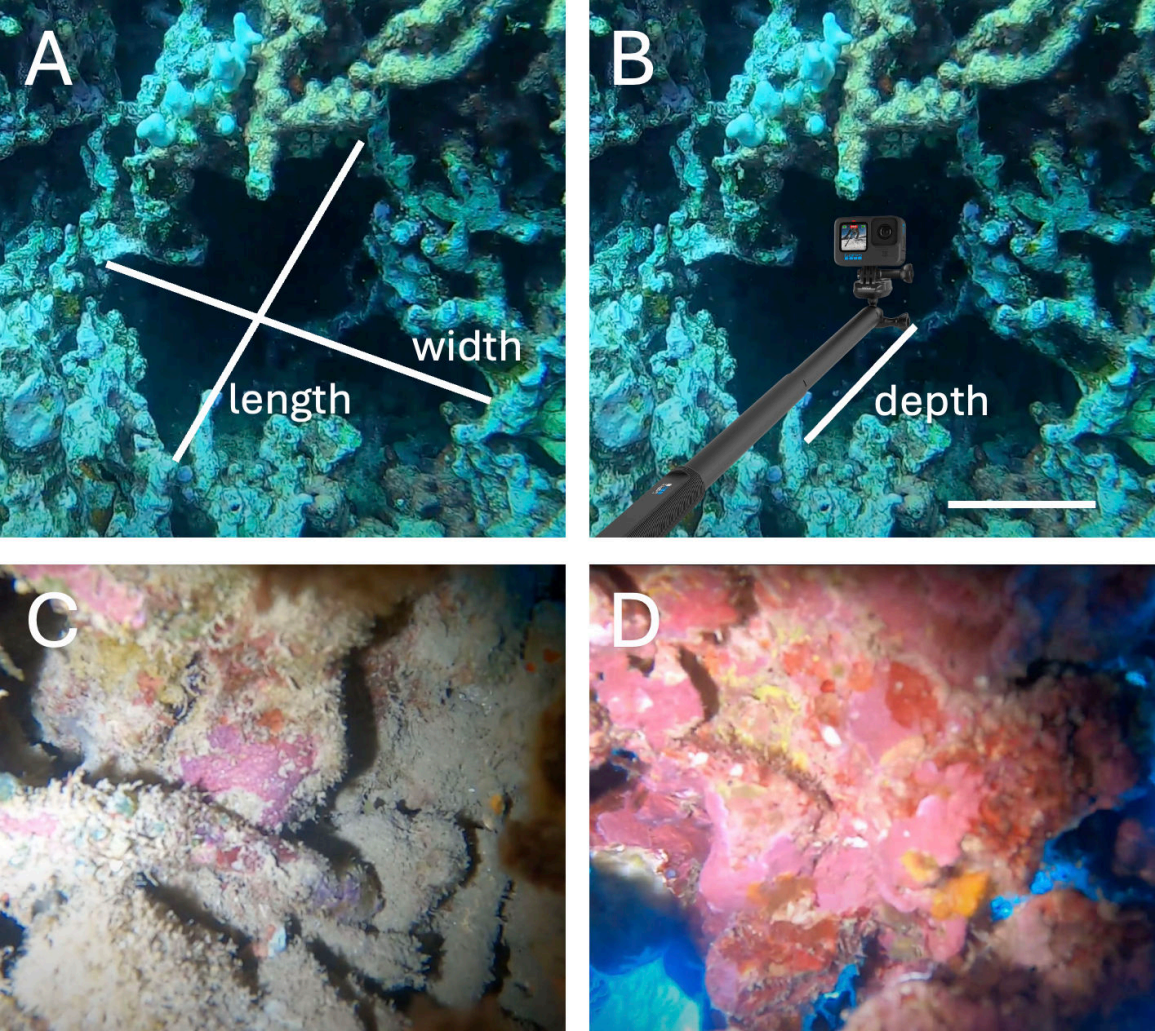
Crevice opening dimensions were measured at (A) the longest length (cm) and widest width (cm) with a ruler. (B) Crevice depth was measured with a ruler on the selfie stick as the furthest distance that the camera could reach into the crevice. Representative screenshots of (C) a crevice wall with high amounts of grey/brown turf algae and sediment laden turf, and (D) a crevice ceiling with high amounts of pink CCA. Scale bar = 10 cm applies only to the crevice dimensions (A and B), as the scale inside crevices is unknown.

Before entering a crevice with the camera set-up, the dimensions of the crevice opening were measured with a ruler in cm as the longest length and widest width of the opening. The maximum length reached by the camera (hereafter referred to as crevice depth) was measured with a ruler in cm on the selfie stick. Thus, the maximum possible crevice depth we could record was 100 cm (the length of the selfie stick). Mean crevice opening size was 27 ± 16 cm (range 8−100 cm) wide and 23 ± 8 cm (range 8−60 cm) long, while maximum crevice depth was 33 ± 15 cm (range 13−100 cm) (figure 2).

Once GoPro videos were recorded, two screenshots per video that represented approximately 20 cm × 20 cm of the crevice were obtained of the back wall and ceiling when the camera was paused at the maximum crevice depth (figure 2). Per cent cover from these screenshots was analysed with point intercept of benthic taxa using Coral Point Count with Excel extensions (CPCe) software that randomly assigned 25 points to each image for visual identification of focal benthic groups (coral, turf algae, CCA) under each point and subsequent calculations of relative per cent cover. Sediment and nine other common benthic functional groups (sponges, red fleshy algae, red calcified algae, green fleshy algae, cyanobacteria, benthic invertebrates, brown calcified algae) were also documented to determine benthic cover in crevices more generally. Owing to the absence of scale bars in the images, only relative per cent cover, and not absolute per cent cover, was analysed.

### 2.3. Abiotic factors and crustose coralline algae abundance

We recorded data on two abiotic factors for each crevice: crevice depth and sedimentation in relation to CCA distribution. Maximum crevice depth was the depth at which the diver recorded the crevice wall using the ruler on the selfie stick as noted above. Sedimentation was estimated from the per cent cover of sediment and sediment-laden turf algae as calculated by CPCe.

To gain preliminary understanding of the relationship between CCA distribution and light, we also recorded light availability in a subset of crevices. Specifically, we revisited one site (KAH2) on 13 January 2023 between 08.14 and 08.46 and measured light availability (in lumens) in 11 individual crevices. We restricted data collection to one day to reduce temporal variation in factors that change hourly and daily, including light availability, cloud cover and turbidity. Specifically, we attached a HOBO pendant temperature and light data logger (Onset Computer Corporation, Bourne, MA, USA) to the selfie stick above the camera such that the light logger floated above the camera and next to the selfie stick, thus preventing light obstruction to the logger by either piece of equipment. As with the camera recordings, relative light readings were taken by inserting the light logger into the crevice to the maximum possible crevice depth for approximately 1 min. Loggers recorded readings every second continuously. The data from the 1 min that the probe was in the crevice was used to calculate average light readings for each crevice and compare relative light (lumens) with the per cent cover of CCA on both the wall and ceiling of the crevice. To ensure accurate comparison of light levels inside the reef, the logger was covered with a duct tape sleeve until divers were prepared to insert the probe into the reef. This, along with noting the time of insertion, ensured that each crevice reading could be clearly distinguished from the data recorded outside of crevices.

### 2.4. Top-reef and crevice crustose coralline algae abundance

To compare the abundance of CCA inside crevices to the well-studied top-reef, per cent cover of the common benthic groups (focal groups: coral, turf algae, CCA; additional functional groups: sponges, red fleshy algae, red calcified algae, green fleshy algae, cyanobacteria, benthic invertebrates, brown calcified algae) was calculated for the top-reef at eight out of the 13 sites in West Maui for which top-reef data were collected simultaneously within the established 10 × 10 m plots described previously (figure 1).

Specifically, top-reef data were collected using large-area imagery described in [39] where a diver swims over the fixed plot using a custom camera rig with two cameras that take continuous images from two angles for future three-dimensional reconstruction of the top-reef. Both cameras had a fixed ISO sensitivity of 400 and fixed focal lengths: one camera had a focal length of 18 mm (wide angle for capturing overlapping imagery) and the other was fixed at 55 mm (higher resolution for species identification). To reconstruct the reef, we used Agisoft Metashape to generate a three-dimensional point cloud of the reef from approximately 5000 underwater photos for each plot using the custom-built software, ‘Viscore’ [48]. For each point cloud, we then assessed per cent cover of benthic taxa using point intercept of benthic taxa with 2500 stratified random points (25 points m^−2^). The raw images used to construct the point cloud were used as a reference for species identification (typically 10−20 images available per point) to ensure high taxonomic accuracy.

Benthic cover of the common benthic groups listed above on the top-reef were compared to those groups on crevice walls and crevice ceilings. Note that the precision of our estimates for benthic cover on the top-reef was probably lower than the precision for estimates inside crevices because the point density for the GoPro images was higher (25 points 20 cm^−2^) than the point density for the top-reef (25 m^−2^).

### 2.5. Turf algae and crustose coralline algae abundance

To examine the relationship between CCA abundance and turf algae as a main CCA competitor, the per cent cover of CCA was compared to the per cent cover of turf algae on crevice walls and ceilings as well as on the exposed top-reef. Specifically, we calculated the ratio of CCA to turf by dividing CCA per cent cover by turf per cent cover. These ratios were calculated separately for each of the eight sites for which top-reef data were available and then pooled to compare the ratios among crevice walls, ceilings and the top-reef.

### 2.6. Statistics

Data were analysed using R (v. 4.4.1, 2024). We transformed the per cent cover data into proportions and constructed a beta regression model (package: betareg) to examine the relationship between relative CCA abundance and the microhabitats inside the reef (crevice ceilings versus walls), crevice depth and the abundance of sediment and sediment-laden turf across sites. As noted previously, sediment and sediment-laden turf abundances were combined and are hereafter referred to as sediment abundance. Including site differences in model construction accounted for both geographical variation and variation in water depth between sites.

To help understand sediment dynamics inside crevices, we also evaluated the relationship between sediment abundance alone and microhabitat, crevice depth and their interactions accounting for site differences using a beta regression model.

We evaluated the relationship between relative CCA abundance and light availability, microhabitat, crevice depth and sediment abundance using a beta regression model for the one site for which we documented light data (KAH2) .

To compare relative CCA abundance of crevice walls and ceilings to that of the top-reef between sites, we first examined differences between CCA abundance in relation to three different coral morphotypes that differed structurally and that dominated the reef at our sites. Specifically, we used the top-reef data to categorize reefs as dominated by branching corals (*Porites compressa*; sites: KEA, HONO1, OLO2), massive corals (*Porites lobata*; sites: KAH1, UKU, WAH) or encrusting/plating corals (*Montipora* spp.; sites: MOLOS, OLO3). We then determined whether there were effects of coral morphotype and habitat type (top-reef, walls, ceilings) on CCA abundance using a beta regression model. Site was not included in the model, as this was a site-level analysis.

To describe turf algae dynamics inside the reef, we examined the relationship between the CCA : turf algae ratio and habitat type (top-reef, walls, ceilings) using a generalized linear model, because there were ratios that were greater than 1.

Finally, to examine community structure, we used a non-metric multi-dimensional scaling (NMDS) analysis to visualize differences in CCA per cent cover in relation to other benthic groups between the top-reef and crevice walls and ceilings.

## 3. Results

### 3.1. Abiotic factors and crustose coralline algae abundance

CCA abundance was examined in relation to crevice microhabitat (ceilings, walls), sediment abundance, crevice depth and their interactions, while accounting for site differences. We found that CCA abundance was higher on crevice ceilings (mean ± s.d. = 31.70% ± 18.42%, range = 19%–53%) compared to walls (15.96% ± 13.59%, range = 8.0%–28%) (table 1). This model also revealed a significant inverse relationship between CCA abundance and sediment abundance (table 1). There were also significant interaction effects on CCA abundance between microhabitat and sediment, microhabitat and crevice depth, as well as between all three of these factors together (table 1).

**Table 1.** Best fit beta regression model results, including the slope estimate ± s.e., the *z*-value and the *p*-value for each estimate, show the effects of microhabitat (relative to crevice walls), sediment abundance, crevice depth, site and their interactions (excluding site) on CCA abundance inside crevices (log-likelihood = 218.6, degrees of freedom = 22). (*n* = 250 crevices. * ⍺ = 0.05, ** ⍺ = 0.005, *** ⍺ = 0.0005.)

factors	slope estimate ± s.e.	*z*-value	*p*‐value
crevice wall	−1.25 ± 0.31	−3.98	<<0.0001***
sediment abundance	−2.87 ± 0.46	−6.19	<<0.0001***
crevice depth	−0.009 ± 0.005	−1.87	0.06
interactions			
crevice wall × sediment	1.71 ± 0.63	2.70	0.007*
crevice wall × crevice depth	0.03 ± 0.01	3.23	0.001**
sediment × crevice depth	0.003 ± 0.01	0.25	0.80
crevice wall × sediment × crevice depth	−0.04 ± 0.02	−2.20	0.03*

To describe sediment abundance, we evaluated the relationship between sediment, microhabitat and crevice depth, while accounting for site differences. In contrast with CCA abundance, we found that there was 29.63% ± 25.43% more sediment on crevice walls (60.80% ± 25.51%, range = 0%–100%) compared to ceilings (31.17% ± 21.89%, range = 0%–92%) and that sediment increased with crevice depth. There were also significant differences in sediment abundance between sites (electronic supplementary material, table S1 and figure S1).

Examining CCA abundance in relation to light availability, microhabitat, sediment abundance and crevice depth revealed that light was not correlated with CCA abundance for this small dataset (mean ± s.d. = 5.10 lumens, range = 0.61–21.25 lumens, *n *= 11 crevices). Interestingly, the only factor that was correlated with CCA abundance was microhabitat, which again showed that CCA abundance was significantly higher on crevice ceilings compared to walls (electronic supplementary material, table S2 and figure S2).

### 3.2. Top-reef and crevice crustose coralline algae and turf abundance

**Figure 3.**
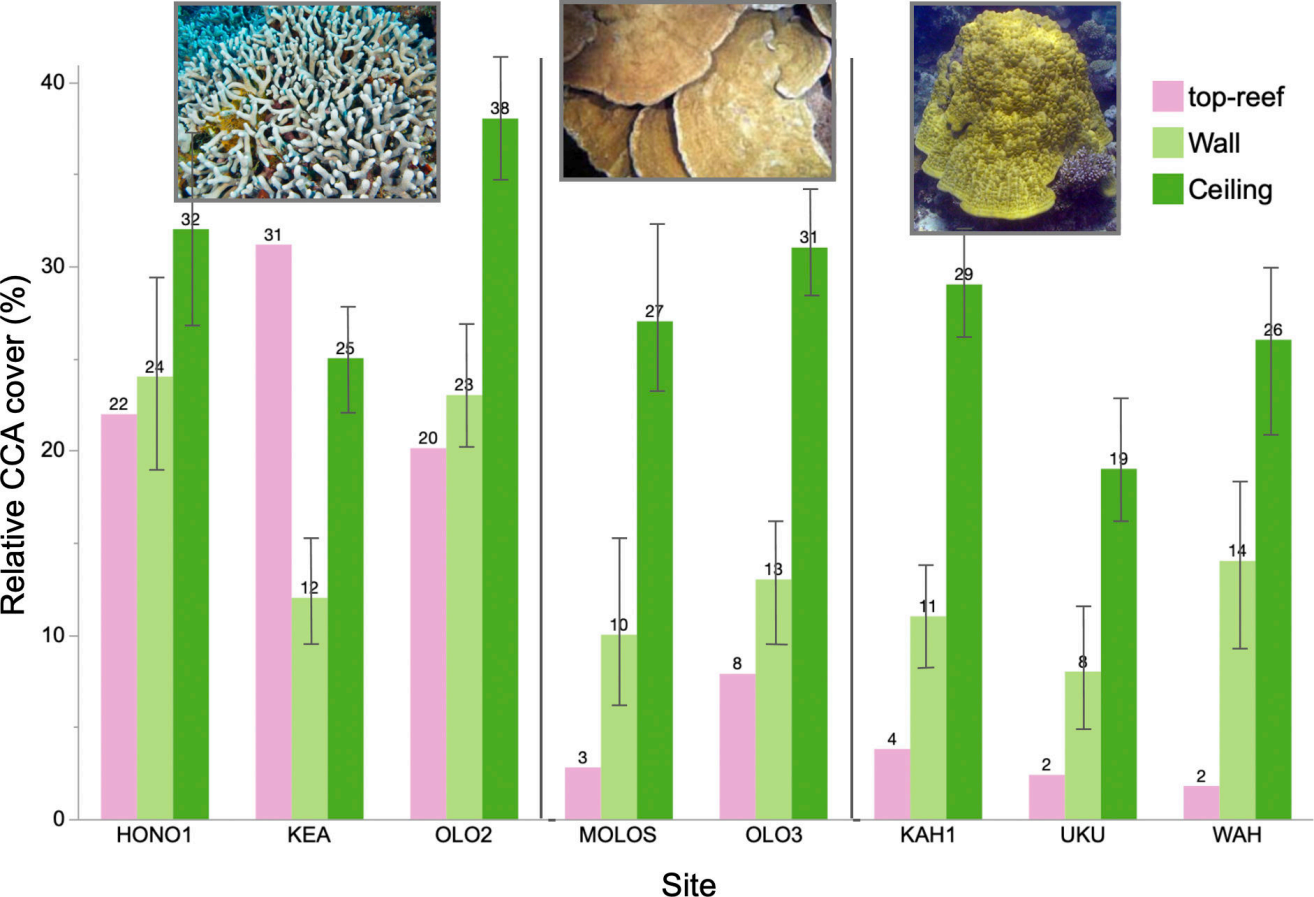
Relative CCA per cent cover across sites dominated by branching (*Porites compressa*; sites: HONO1, KEA, OLO2), plating (*Montipora* spp.; sites: MOLOS, OLO3), and massive (*Porites lobata*; sites: KAH1, UKU, WAH) coral species. Sites dominated by branching coral species appeared to have higher CCA cover overall than reefs dominated by plating or massive corals. All sites had higher CCA per cent cover on crevice ceilings (dark green) than on the walls (light green) and top-reef (pink) except for KEA. Sites are listed from north to south on West Maui. Error bars = standard deviation. Pink bars for the top-reef do not have error bars, because CCA abundance was quantified for the entire 10 x 10 m plot at each site.

Of the eight sites for which we were able to quantify CCA per cent cover on the top-reef, in addition to the crevice walls and ceilings, all but one (KEA) exhibited higher CCA abundance inside the reef than on the top-reef. Crevice ceilings had more CCA (mean = 36.03% ± 6.73%) compared to crevice walls (mean = 17.72% ± 6.83%) and the top-reef (mean = 11.61% ± 11.44%; table 2; figure 3). To examine coral composition in relation to CCA per cent cover in crevices, data were grouped by dominant top-reef coral morphotypes (massive, plating, branching; figure 3). Given the small sample size of eight sites, we examined the relationship between CCA abundance, habitat and coral morphotype only. While there were no significant differences in CCA abundance between the coral morphotypes (mean ± standard deviation: branching = 28.28% ± 8.90%, massive = 17.16% ± 14.75%, plating = 19.01% ± 15.17%), there were interaction effects on coral abundance with regards to the top-reef and massive and plated corals (table 2).

**Table 2.** Best fit beta regression model results, including the slope estimate ± s.e., the *z*-value, and the *p*-value for each estimate, show the effects of habitat type (relative to crevice walls), coral morphotype (branching, plating, massive) and their interactions on CCA abundance inside crevices (log-likelihood = 41.89, degrees of freedom = 10). (*n* = 24. Only significant interactions effects are shown. ** ⍺ = 0.005, *** ⍺ = 0.0005.)

factors	slope estimate ± s.e.	z-value	*p*‐value
top-reef	−0.60 ± 0.22	−2.79	0.005**
crevice wall	−0.76 ± 0.22	−3.43	0.0006**
massive coral	−0.20 ± 0.21	−0.98	0.33
plating coral	0.02 ± 0.23	0.07	0.95
interactions			
top-reef x massive coral	−2.10 ± 0.44	−4.72	<<0.0001***
top-reef x plating coral	−1.77 ± 0.45	−3.92	<<0.0001***

**Figure 4.**
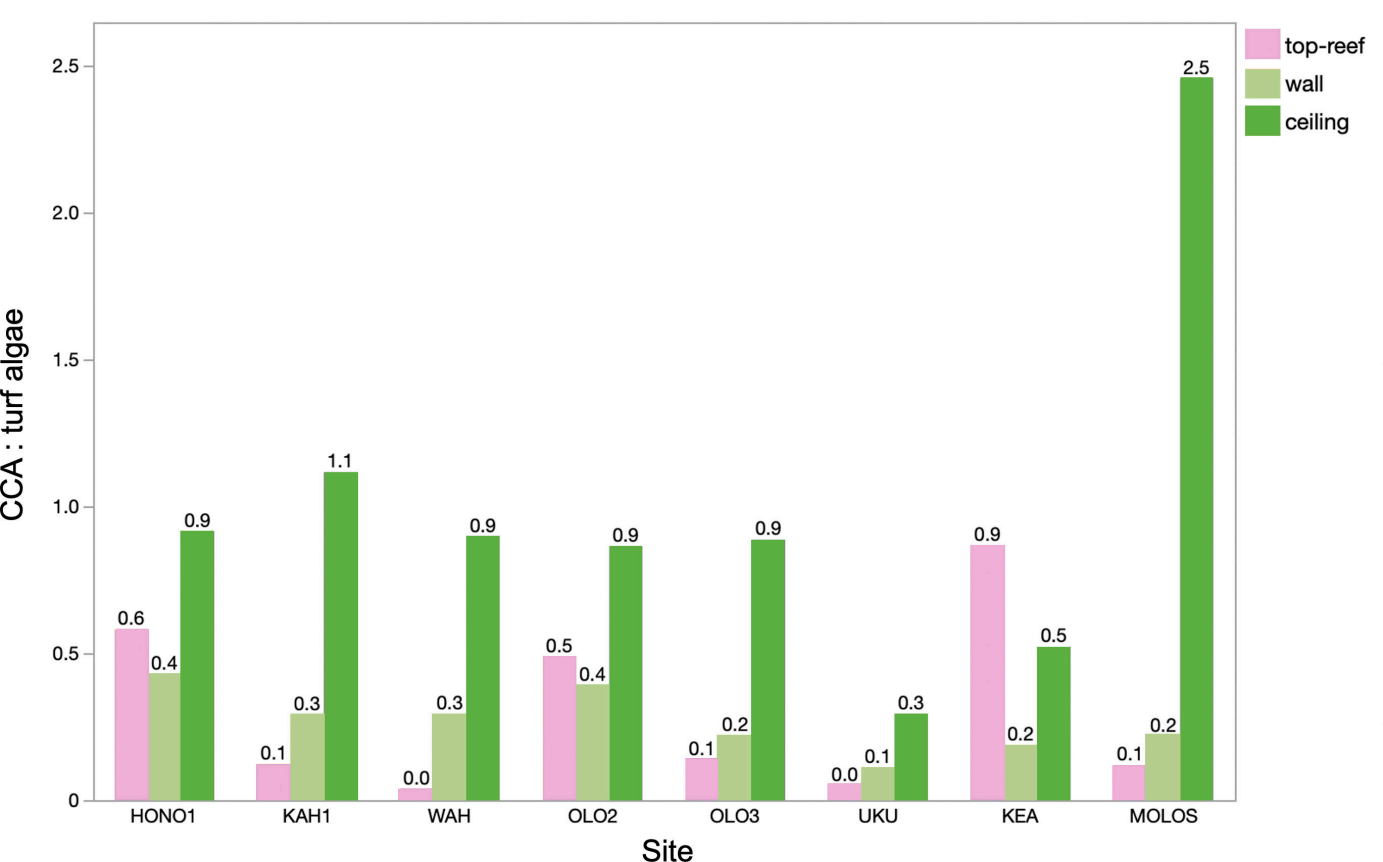
CCA : turf algae ratio shows higher CCA cover compared to turf algae on the top-reef (pink), wall (light green) and ceiling (dark green), with the ceiling having significantly higher ratios compared to the wall and top-reef. Sites listed from north to south on West Maui.

To describe sediment and turf algal dynamics inside the reef, we examined the relationship between the CCA : turf algal ratio and habitat using a generalized linear model (as opposed to a beta regression model). The mean ± standard deviation CCA : turf algae ratio for crevice ceilings (1.33 ± 1.02), was significantly higher than the mean ratios of the walls (0.33 ± 0.14), and top-reef (0.30 ± 0.31; electronic supplementary material, table S3). The CCA : turf ratio was almost three times greater at Molokini Shallow (MOLOS) than at any other site (figure 4). MOLOS is one of the furthest offshore sites and is located inside a no-take marine reserve, and thus it is considered one of the healthier top-reef systems of those surveyed. KEA was the only site that had a higher CCA : turf ratio on the top-reef than on crevice ceilings and walls (figure 4).

**Figure 5.**
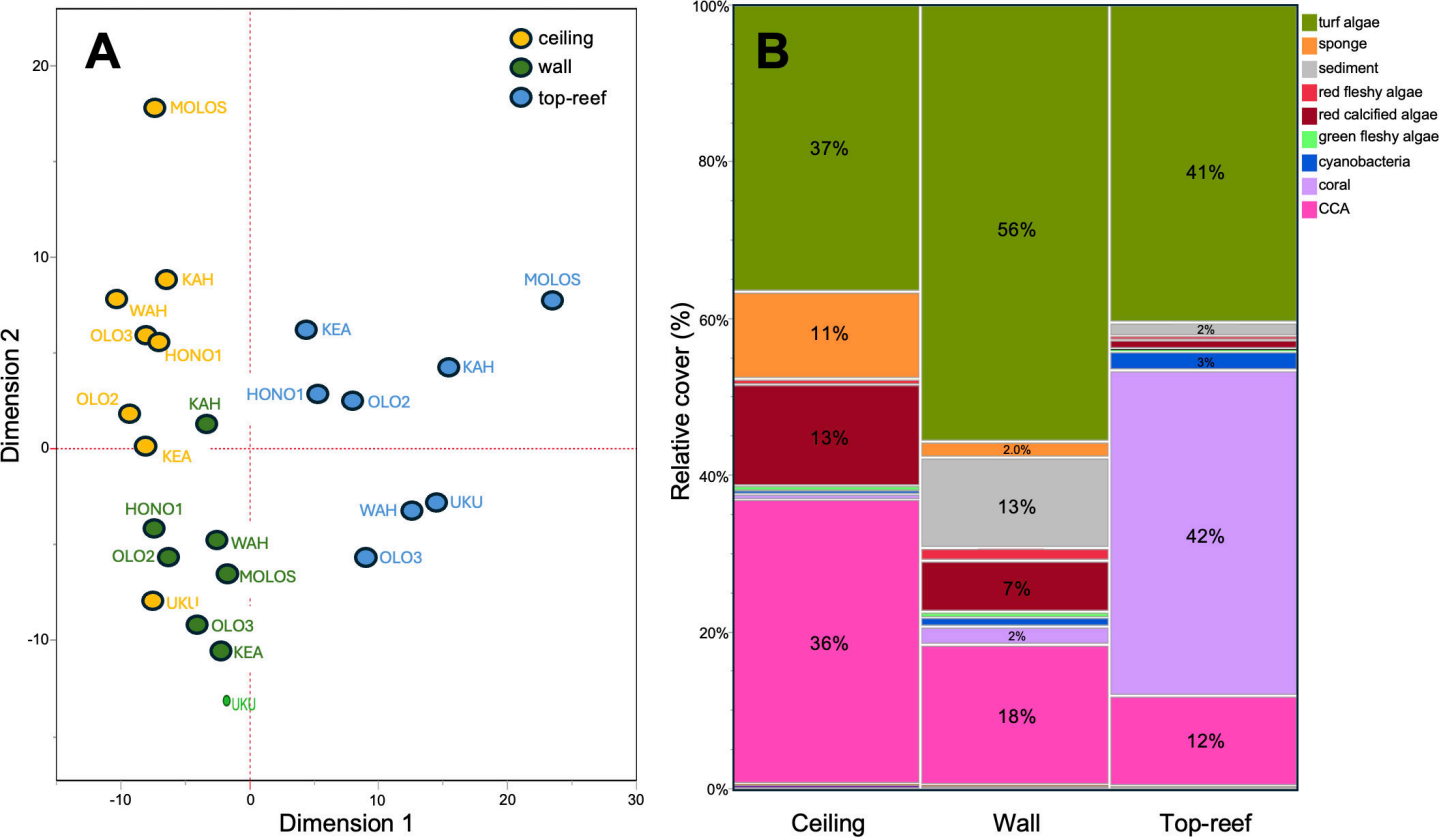
(A) NMDS plot of species composition among sites with data for the crevice ceilings (yellow dots), walls (green dots) and top-reef (blue dots) illustrate substantial differences in community structure between these habitats. (B) Mosaic plot of CCA and turf along with eight other functional groups shows that coral cover may be contributing to differentiation on the top-reef, whereas CCA and sponge cover yield differentiation inside crevices.

Data visualization with NMDS further showed separation in community structure of the top-reef, crevice walls and crevice ceilings (figure 5A). A mosaic plot with the diversity of organisms that we quantified in the community demonstrated that coral cover appears to contribute to differentiation of the top-reef, while CCA and sponge cover contribute to the differentiation between crevice walls and ceilings (figure 5B). Very few corals were found in crevices, and those observed often resulted from coral extensions from top-reef colonies. On occasion, small coral recruits were anecdotally observed near crevice openings, but low densities of coral recruits is not uncommon on reefs in Hawai‘i [43,44].

## 4. Discussion

Using relatively inexpensive, off-the-shelf GoPro cameras, we provide the first *in situ* estimates of CCA per cent cover in crevices. We show that CCA cover is higher inside the reef compared to the top-reef. Combined CCA per cent cover on crevice walls and ceilings ranged from 27–61%, which is extremely similar to CCA cover measured from (non-*in situ*) CAUs (27%–62%) [28]. To roughly compare the surface area of CCA cover inside the reef with that of the top-reef, we approximated crevice surface area using our measurements of crevice opening length, width, and depth. We found that CCA cover inside reefs is 1.1−5.6 times greater than on the top-reefs across our eight sites where top-reef data were available. Thus, the relatively high coverage of CCA inside the reef suggests that estimates of sediment stabilization and calcium accretion are likely also higher than previously documented [16,28,29]. Inside crevices themselves, we consistently found that ceilings and walls were two distinct microhabitats and that CCA cover was highest on ceilings near crevice openings where sediment cover was the lowest. These findings provide a more fine-scale analysis of CCA abundance and distribution and further our understanding of where in the reef net accretion may originate.

### 4.1. Sedimentation and turf algae abundance correlate with crustose coralline algae cover

Across our analyses, CCA abundance was highly correlated with sediment abundance. These results mirror the exposed reef on large scales, whereby CCA abundance typically peaks on the outer shelf and decreases towards the inner shelf because of higher sedimentation loads in shore [34]. Inside crevices, sediment has been estimated to dominate 40% of crevice substrate overall [49]. This estimate more closely matches our findings for crevice ceilings (mean = 31% ± 22% across all sites) but is lower than our findings for crevice walls (mean = 61 ± 26%), which could suggest an underestimate of sediment cover in previous research. Our findings further indicate that crevice ceilings provide more suitable habitat for CCA than crevice walls, because ceilings have significantly less sedimentation than walls and CCA cover significantly increases as sediment cover decreases. However, crevices are also considered sinks for sediments on reefs that are high in particulate organic matter (POM) and dissolved organic matter (DOM) [49]. Thus, while crevice ceilings probably provide more suitable habitat for CCA, crevice walls may be the primary reservoirs for POM and DOM. These examples demonstrate that crevice walls and ceilings are separate microhabitats within reefs that may provide different key functions. One additional microhabitat that we did not document was the crevice floor, because we observed the floor to be almost entirely covered in sediment. However, future research that documents crevice floors would help increase the accuracy of sedimentation estimates and therefore further our understanding of sediment dynamics in reefs.

Counter to previous results [50], CCA cover was not correlated with crevice depth. However, CCA cover did significantly correlate with the interactions between depth and microhabitat and depth, microhabitat and sedimentation (table 1). Sediment abundance was also highly inversely correlated with crevice depth. The amount of water flow within the reef has been shown to decrease with crevice depth yielding increased sedimentation within crevices in Curacao [50]. Interactions between water flow and sedimentation were probably occurring within the crevices measured here as well, which suggests multifaceted relationships between water flow, sedimentation, crevice depth and CCA abundance that warrant significant future study.

Light availability also did not correlate with CCA cover at the one site we examined, perhaps because of the small sample size of light data (*n *= 11 crevices). However, fluctuating light availability was similarly observed in eight crevices in Curacao, which explored larger crevices and coral overhangs [50]. These results reflect the overall heterogeneity of crevice openings, their size and the presence of cracks in the reef matrix, which could introduce variation in light availability at different crevice depths.

To examine changes in CCA abundance relative to turf algae, which competes for space with CCA, we calculated the ratio between CCA and turf algae. Crevice ceilings exhibited an almost 1 : 1 ratio between CCA and turf algae at most sites, but turf algae was favoured on both the top-reef and crevice walls (figure 4). Nearly 85% of all sediments found on walls and 99% of sediments found on ceilings were bound by algal turf mats. These findings align with previous research indicating that turfs can bind up to 87% of sediment on the top-reef [38] and illustrates how turf algae can contribute to sediment dynamics inside reefs. Turf algae on crevice walls were especially heavily sediment-laden, and visual assessment could not determine if these turfs were alive or dead. Therefore, it is uncertain whether turf has genuinely adapted to these low light habitats or if low flow rates or low oxygen prevent other benthic species from overgrowing dead sediment-laden turf [36,38]. These findings, combined with the relationships between CCA abundance and sedimentation previously discussed, emphasize the necessity for further experimentation to disentangle the abiotic and biotic factors influencing CCA distribution.

### 4.2. Crustose coralline algae abundance is higher in crevices than on the top-reef

Of the eight sites with CCA top-reef data, all but one had significantly higher CCA cover inside the reef than on the top-reef. Top-reef habitats were mainly dominated by large adult corals and turf algae, both of which outcompete CCA [28,35,51,52]. Crevices were dominated by CCA and turf algae (figure 5). Corals found in crevices were located near openings and were either extensions from top-reef colonies or small recruits. It is worth noting that defining the border between the top-reef and a crevice can be diffuse. Thus, what we defined as a crevice opening may be functionally similar to the top-reef for a coral recruit.

Ceilings had consistently higher CCA abundance than both the top-reef and walls. As mentioned previously, low sedimentation is related to higher CCA cover on crevice ceilings. Additionally, there are probably fewer benthic groups competing for space with CCA inside crevices, especially on ceilings. For example, macroalgae, another significant competitor of CCA [18] and an observed group on the top-reef at our sites, was rarely documented inside crevices, potentially leaving more available habitat for CCA.

Grouping sites by top-reef coral morphotype showed that there was no significant effect of morphotype on CCA abundance alone but that there was an interaction effect between habitat and morphotype implying that crevices in branching coral reefs have higher CCA abundance (table 2; figure 3). For example, HONO1 and OLO2 had the highest CCA per cent cover inside the reef (56% and 61%, respectively), and all three branching coral sites also had the highest overall top- and internal-reef CCA cover (HONO1: 78%, KEA: 68%, OLO2: 81%; figure 3). Branching corals themselves have faster bioerosion rates compared to massive and plating corals, and faster erosion leads to more coral rubble, which tends to be heavily colonized by CCA [53,54]. They also have increased spaces for cryptobenthic herbivores, including urchins and fishes, to access turf algae thereby creating more space for CCA settlement [54–56]. Thus, branching coral habitats may be more conducive to CCA growth. Interestingly, unlike all other sites, KEA had substantially higher top-reef CCA cover than CCA cover on crevice walls and ceilings. Because of the high structural complexity of branching coral at this site, KEA lends itself to higher abundances of cryptobenthic herbivores seeking predation refuge [55,56]. These grazers would also consume CCA competitors, such as fleshy algae, leaving more space for CCA growth.

These findings highlight how coral morphology can impact reef structural complexity, influencing not only individual crevices but also the broader reef landscape across a range of ecological scales. Previous research in Hawai‘i has found that different coral morphotypes have different structural signatures, with branching *Porites* corals creating the most three-dimensional habitat structure at the scales considered in this study, followed by massive *Porites* and lastly by encrusting *Montipora* [40]. The variability in CCA abundance we observe here may follow the variation in structural complexity of the dominate reef morphotypes, because the shapes and sizes of reef crevices would be expected to vary based on the growth form of the dominant coral on a reef. Beyond the coral community, each site is characterized by a complex interplay of biotic and abiotic factors as well as local human impacts that contribute to site-specific variation in community composition and structural complexity, both on the top-reef and within the reef [39,57].

### 4.3. Low-cost methods yield new insights into crustose coralline algae abundance inside reefs

The knowledge that CCA fulfils important ecological functions on coral reefs has come primarily from studies of the top-reef [4–7]. Previous diversity studies on the top-reef have highlighted the need to transition from two-dimensional to three-dimensional monitoring of reefs to accurately quantify ecologically important groups, such as CCA, that are abundant on vertical surfaces like coral skeletons but are often under-represented in two-dimensional analyses [4]. While certainly an improvement over two-dimensional analyses, large-area imaging techniques (as typically practised) do not account for areas of the reef that are occluded from the viewpoint of a diver taking top-down photographs. Our approach using GoPro cameras allowed us to capture these previously inaccessible areas and provide a more comprehensive view of the roll of CCA in the reef ecosystem.

Artificial structures that simulate cryptic spaces, including CAUs and autonomous reef monitoring structures (ARMs), represent an increasingly popular and standardized approach that has significantly advanced our understanding of cryptic diversity, because they allow for the monitoring of recruitment of benthic communities over time [6,7,24,27]. However, these methods typically require 2−3 years of community settlement in the field. Moreover, they may inadvertently overlook ecological patterns that emerge within much older, natural reef communities, as demonstrated in this study. GoPros are inexpensive and widely available to researchers and even citizen scientists and can therefore greatly help expand knowledge of broadscale species distribution patterns inside reefs.

## 5. Conclusions

Overall, our findings using low-cost GoPros to explore inside coral reefs revealed relatively high abundances of CCA in cryptic habitats when compared to the extensively studied top-reef. Given that the surface area of CCA inside reefs potentially exceeds the surface area of the top-reef by 1.1−5.6 times, this research not only demonstrates the large abundance of CCA inside the reef matrix, but it also underscores the importance of studying the functional role of cryptic habitats to understand holistic reef functioning. For example, high CCA coverage with low algal and sediment coverage on the top-reef form coral recruitment habitats [58,59]. Given that the internal reef probably has high CCA coverage, CCA may be contributing more to coral recruitment than is currently documented [51]. Yet, CCA is a relatively diverse group that has been shown to either promote or inhibit coral growth [51]. CCA are difficult to identify based on morphology alone, but future research examining CCA species composition would help untangle these different functional roles inside reefs.

Crevices could also serve to promote CCA growth and therefore reef accretion overall but especially in response to environmental disturbances, such as storms, coral bleaching events and ocean warming and acidification [24,60,61]. Crevices are low-light habitats with minimal water flow that trap excessive respiration from cryptic species, leading to a more acidified, lower oxygen microenvironment that could reduce overall calcium accretion [62,63]. However, given the ability of CCA to grow in disturbed habitats, crevices may be central to the ability of reefs to tolerate and recover from increasingly frequent disturbances. For example, while the negative impact of ocean acidification on calcifiers is widely acknowledged on the top-reef [57,64], intriguingly, experiments with ARMS revealed shifts in cryptobenthic communities towards higher abundances of encrusting red algae, including CCA, under combined ocean warming and acidification conditions [65]. These findings, in conjunction with our own observations of high CCA per cent cover in crevices, raise the possibility that cryptic CCA is potentially adapted to naturally lower pH environments and may exhibit resilience to the adverse effects of future ocean acidification, unlike their top-reef counterparts. Future studies documenting flow rates, pH and oxygen inside reefs as well as variation in calcification rates between cryptic and non-cryptic CCA (similar to [28]) would provide insight into the adaptive capacities of cryptobenthic communities to disturbances and the impact that community shifts may have on ecological function.

In the next 20−30 years, the detrimental effects of climate-driven disturbances, such as more frequent and intense storms, rising ocean temperatures and ocean acidification, are predicted to exacerbate reef degradation [66]. Consequently, the loss of habitat structure and the overall flattening of reefs will likely reduce the number of cryptic habitats available for CCA growth and the various other species that rely on these spaces [67]. Our results support the importance of cryptic habitats for maintaining coral reef functioning and reveal the need to uncover information about cryptic habitats that will be critical for understanding future coral reef growth, maintenance and resilience.

## Data Availability

Data for this study is publicly available through Dryad [68]. Supplementary material is available online [69].
